# Starvation During the Larval Stage Driving Population Decline in the Butterfly Specialist *Luehdorfia chinensis* Leech, 1893 (Lepidoptera: Papilionidae)

**DOI:** 10.3390/insects16100995

**Published:** 2025-09-24

**Authors:** Wenjing Yang, Qi Zhu, Yunhao Zou, Chao Yang, Wenguo Wu, Qin Zou, Juping Zeng

**Affiliations:** 1 Jiangxi Provincial Key Laboratory of Conservation Biology, Key Laboratory of National Forestry and Grass and Administration on Forest Ecosystem Protection and Restoration of Poyang Lake Watershed, College of Forestry, Jiangxi Agricultural University, Nanchang 330045, China; yangwenjing9351@163.com (W.Y.);; 2 Lushan Forest Ecosystem Observation Station, Lushan National Nature Reserve of Jiangxi, Jiujiang 332900, China; 3 Lushan Forestry Bureau of Jiangxi, Lushan 332800, China; 4 Taohongling Sika Deer National Nature Reserve of Jiangxi, Pengze 332724, China

**Keywords:** *Luehdorfia chinensis*, larval starvation, host plant limitation, population dynamics, life table

## Abstract

Host plant limitation poses a major threat to the endangered specialist butterfly *Luehdorfia chinensis* Leech, 1893, whose larvae are oligophagous at the species level on *Asarum* spp., while local populations often appear monophagous, depending on the host plants (*A. sieboldii* Miq. or *A. forbesii* Maxim.) available in their habitat. To simulate natural starvation caused by host plant scarcity, third- to fifth-instar larvae were subjected to a three-day deprivation treatment, and the effects on individual fitness traits—including larval development, pupal duration, and adult fecundity—were assessed, along with population dynamics. Starvation significantly prolonged larval development, shortened the pupal stage, reduced female fecundity, and markedly decreased key population parameters, such as the intrinsic rate of increase (*r_m_*) and the net reproductive rate (*R*_0_). Population projections further indicated that repeated starvation stress could reduce population size by more than 83% within two years, potentially intensifying genetic drift, inbreeding depression, and demographic instability, ultimately increasing the risk of extinction. These findings provide direct evidence that host plant limitation drives population decline in *L. chinensis*, contribute to the broader understanding of global butterfly declines, and underscore the critical importance of conserving and restoring essential habitat resources. Moreover, they highlight the relevance of the resource-based habitat concept for the effective protection of specialist species.

## Simple Summary

Specialist butterflies, exemplified by the endangered *Luehdorfia chinensis* Leech, 1893, in China, depend on a small range of host plants for survival. Scarcity of these host plants can expose larvae to short-term starvation, negatively affecting their growth, survival, and reproduction. Here, we simulated a three-day starvation treatment in third- to fifth-instar larvae under laboratory conditions and evaluated its effects on larval development, pupal duration, adult longevity, and fecundity. Age-stage, two-sex life table analysis was further applied to model population dynamics under starvation stress. Short-term starvation significantly prolonged larval development, shortened pupal duration, reduced female fecundity, and substantially decreased population growth potential. Population projections indicated that repeated host plant scarcity could drive declines exceeding 83%, thereby elevating the risks of inbreeding and local extinction. These findings highlight the urgent need to conserve and restore host plant resources for specialist butterflies, providing crucial guidance for the long-term conservation of endangered insect species.

## 1. Introduction

In recent decades, with the intensification of global climate change and the increasing frequency of human disturbances, global biodiversity is facing unprecedented pressure [1,2,3], and many taxa have shown declining diversity levels [4,5]. Butterflies, as one of the most sensitive indicators of environmental changes [6,7], show evidence of substantial global declines, which may be more apparent due to the availability of long-term monitoring data. Long-term monitoring data from various regions consistently indicate that butterflies are undergoing population declines [8,9,10], the situation being even more severe in specialist species [11].

Butterfly decline is a complex ecological process driven by multiple factors, including natural enemies [12,13], climate change [14], and habitat degradation. As for monophagous or oligophagous butterfly species, host plant availability and limitation are among the key factors influencing population trends [15,16,17,18]. Primarily, two types of ecological pressure could be imposed by the availability of host plants in shaping larval dispersal and foraging behaviors: (1) density-dependent resource limitation, where larvae experience stress when the number of host plants per unit area is insufficient to support normal feeding; and (2) increased search costs due to greater distances between host plant patches. These two pressures jointly determine the spatial distribution patterns and local population occurrence in herbivorous butterflies [19,20]. Host plant limitation not only increases the difficulty and risk of oviposition site selection for females [21,22] but also forces larvae to disperse more frequently during development, thereby increasing their exposure to starvation stress and predation risk [15,23], ultimately potentially disrupting population establishment and persistence. Therefore, it is important to investigate the decline of monophagous butterflies by directly assessing the effects of starvation—resulting from host plant limitation—on individual fitness and population development [12].

*Luehdorfia chinensis* Leech, 1893, is a monophagous butterfly endemic to China, with a narrow range primarily in mountainous and hilly areas of the Qinling Mountains and the middle to lower reaches of the Yangtze River [24,25,26]. Its larvae feed exclusively on the perennial herbs *Asarum forbesii* Maxim. and *A. sieboldii* Miq. [27,28]. These host plants are shade-tolerant perennial herbs that usually grow in the understory of moist forests and shrublands [29], sprouting in early spring to coincide with butterfly emergence and providing substrates for oviposition and larval food resources. However, due to their long-standing medicinal value, intensive harvesting has led to severe patch fragmentation and population decline in many regions [30]. The distribution and survival of *L. chinensis* populations are highly sensitive to host plant limitation, including both spatial configuration (e.g., patch distance) and quantitative characteristics (e.g., host plant abundance) of patches [23,31,32]. In particular, once larvae develop to the third instar, they abandon gregarious feeding behavior and begin to disperse individually into surrounding patches. During this stage, their success in locating food—as well as the associated time, energy expenditure, and risks (e.g., starvation, predation)—is largely determined by the spatial pattern of host plant patches, including patch size, spacing, and density [12,33]. For example, in Taohongling of Jiangxi Province, *L. chinensis* has only been recorded in two relatively large *A. forbesii* patches—Maoyingwo [23,34] and Taohongsha [32,35]—underscoring its strong dependence on large, continuous host plant patches. This dependence likely influences larval survival, as some individuals are forced to disperse over long distances due to low host plant encounter rates (e.g., low density or scattered distribution), thereby facing elevated risks of starvation and predation that may reduce fitness (e.g., slower development) or result in mortality [31,32].

Importantly, the specific host plants of *L. chinensis* (*A. forbesii* and *A. sieboldii*) are of high medicinal value and have long been harvested for use in traditional Chinese medicine [36,37]. Overharvesting has led to severe fragmentation of host plant patches across many regions, causing drastic declines in plant abundance and even local extinctions [35,38,39]. Under such conditions, *L. chinensis* has likely experienced local extinctions [25], such as the population in Lushan, or drastic reductions in population size, placing its long-term persistence at risk [32]. Recognizing its high conservation value, *L. chinensis* was listed on the IUCN Red List as early as 1986 and designated as a Class II species in the Chinese National Key Protected Wild Animals List in 1989 [40]. Therefore, it is urgent to clarify the population dynamics of this rare butterfly under host plant limitation, identify strategies to mitigate its decline, and develop approaches for population recovery through host plant restoration. This study aims to investigate butterfly fitness (developmental duration, survival rate, reproductive capacity, etc.) under starvation stress during the dispersal larval stage (third to fifth instars), simulating the effects of host plant limitation on the occurrence of *L. chinensis* in Taohongling.

## 2. Materials and Methods

### 2.1. Insect Sources

In March 2021, mature eggs of *L. chinensis* were obtained from the Conservation Research Base of Taohongling, Jiangxi Province, China. The eggs were kept at ambient room temperature in Taohongling. Egg hatching was monitored daily. The newly hatched larvae were then individually transferred to transparent plastic containers for rearing and observation.

The Conservation Research Base is located at an elevation of 410 m, within the Taohongling Sika Deer National Nature Reserve region. The reserve has a subtropical monsoon climate, with an average annual temperature of 16.5 °C, annual precipitation of 1172 mm, and annual evaporation of 1587.2 mm [41].

### 2.2. Larval Rearing and Starvation Treatments

In Taohongling, the butterfly larvae of *L. chinensis* feed exclusively on leaves of *A. forbesii.* Therefore, fresh leaves with petioles were collected to rear larvae in experiments. The leaves were inserted into moist putty after being rinsed with clean water and placed in transparent containers (17.2 cm in diameter, 13.2 cm in height). Then, the newly hatched larvae were transferred to leaves using a fine brush. Each larva was reared individually. During the 1st–2nd instars, a fresh leaf was replaced only if the previously supplied leaf had wilted. At the 3rd instar, one fresh leaf was added on average every two days, and during the 4th–5th instars, one fresh leaf was added daily. All leaves used for feeding had an area exceeding 35 cm^2^. The containers were placed at ambient room temperature (see Supplementary Figures S1 and S2). During rearing experiments, larval droppings and remaining host plant debris were removed daily, and the containers were washed with tap water, disinfected with 75% ethanol (Sinopharm Chemical Reagent Co., Ltd., Shanghai, China), and air-dried before reuse.

Field observations reveal that the larvae of *L. chinensis* will leave the original host plant stem once they molt into the third instar, which indicates the larvae shift to the actively dispersal stage for searching for more host plant resources needed. However, the risk of starvation stress could increase if facing hostplant limitation. Therefore, to simulate this situation, the third-instar or older larvae were used in starvation experiments (SE). According to the method of Guo [42], newly molted larvae were returned to normal rearing after undergoing three days of starvation (without causing mortality). As such, there were three starvation treatments: the third-instar, fourth-instar, and fifth-instar starvation groups, and the control group (CG, without starvation stress) in this study. Each group used 30 larval individuals. Once they ceased feeding and reached the pre-pupal stage, the fifth-instar larvae were transferred to rearing cages for pupation. The pupae were labeled and kept in cool, dark conditions until emergence the following year. Throughout the rearing experiments, larval instars, survival status, and other parameters were recorded daily, and the developmental duration and other life history parameters were calculated accordingly.

### 2.3. Adult Mating and Oviposition

In early March 2022, overwintered pupae from the same group were transferred to rearing cages for the purpose of adult emergence. The cages contained a potted nectar plant (*Viola philippica* Cav.), serving as a natural floral resource, and cotton balls soaked in honey water to provide an additional, easily accessible energy supplement. The emergence time and sex of each butterfly were recorded daily. Pairs of newly emerged adults from the same treatment group were then transferred to separate mating cages, which contained the same nectar plant and honey-soaked cotton balls, as well as a potted host plant (*A. forbesii*) to allow oviposition. The mating time, individual labels, survival, egg production, and other parameters were observed and recorded daily. During the observation, water was periodically sprayed inside the cages to maintain appropriate environmental humidity.

### 2.4. Data Analysis

#### 2.4.1. Life Table Data Analysis

The age-stage, two-sex life table approach was used to analyze the raw life-history data for *L. chinensis* under control and starvation treatments [43,44]. The age-stage-specific survival rate (*s_xj_*), defined as the probability that an individual of age *x* and stage *j* survives to that specific stage, was evaluated. The age-stage-specific fecundity (*f_xj_*), representing the daily number of eggs laid by an individual at age *x* and stage *j*, the age-specific fecundity (*m_x_*), and the age-specific survival rate (*l_x_*), referring to the probability that a newly laid egg survives to age *x*, were calculated accordingly [44,45,46].

The intrinsic rate of increase (*r*) was calculated based on the Euler–Lotka equation $\sum_{x\;=\;0}^{\text{∞}}{e^{-r\left(x\;+\;1\right)}l}_{x}m_{x}\;=\;1$ [47,48]. The finite rate of increase (*λ*) was computed as *λ* = *eʳ* [49]. The net reproductive rate (*R*_0_) was estimated as $R_{0}=\sum_{x=0}^{\infty }l_{x}m_{x}$ [50]. The mean generation time (*T*), defined as the time required for the population to increase *R*_0_-fold under a stable age-stage distribution, was calculated as *T* = ln(*R*_0_)/*r* [51]. The gross reproductive rate (GRR) was calculated as GRR = ∑*m_x_* [46].

The raw life-history data were entered into Microsoft Excel 2013 (version 15.0), and the TWOSEX-MSChart^®^ software (version 2020.05.28; available at https://lifetablechi.com/software/ (accessed on 22 March 2024)) was used for all life table analyses [52]. This program provides a simplified and standardized method for computing population parameters, avoiding the complexity of manual calculation. The means, standard errors, and variances of the population parameters were estimated using the bootstrap method (with 100,000 replications), which is integrated into the TWOSEX-MSChart^®^ software [52]. All graphs were produced using Origin 2022 (version 9.9.0, *OriginLab*, Northampton, MA, USA).

#### 2.4.2. Population Projection

To predict and compare the population growth and age-stage structure of *L. chinensis* under control and starvation treatments, we used life table data on developmental time, survival rate, and fecundity to simulate population growth using the TIMING-MSChart program (version 2020.03.31) [52]. The data file for TIMING-MSChart (version 2020.03.31) was generated directly from the output file “15_For_TIMING.txt” produced by TWOSEX-MSChart (version 2020.05.28), simplifying the data preparation process [53].

To assess stage-specific growth dynamics, the stage size at time *t* was calculated as log_10_(*N_j,__t_* + 1), and the daily growth rate of stage *j* from time *t* to *t* + 1 was computed as φ_j,t_ = ln (*N_j,t+_*_1_ + 1) − ln (*N_j,t_* + 1). This method avoids undefined values caused by zero individuals at a given time point and allows consistent comparisons across treatments [54]. For statistical analyses, differences between two independent groups (CG vs. combined 3rd–5th instars) were evaluated using the Mann–Whitney U test, and differences among the four independent groups (CG, 3rd–5th instars) were evaluated using the Kruskal–Wallis test followed by Dunn’s post hoc test. Statistical significance was set at *p* < 0.05.

## 3. Analyses and Results

### 3.1. Effects of Starvation on Developmental Duration and Survival

Starvation significantly influenced the larval developmental duration of *L. chinensis* (measured in days, d). As shown in Table 1, the durations were extended by 32.4% (5th-instar starvation group) to 103.9% (3rd-instar starvation group) relative to the control group (*χ*^2^ = 99.97, *df* = 3, *p* < 0.0001). Although subsequent instars of the starvation groups showed durations similar to or shorter than those of the control group. Overall, the larval period remained significantly prolonged, with the 3rd-instar group exhibiting the most pronounced delay. In contrast, Starvation significantly shortened pupal developmental duration (*d*) (*χ*^2^ = 9.83, *df* = 3, *p* < 0.05; Table 1), indicating that the butterfly emergence date would occur earlier than that of non-starved individuals.

**Figure 1:**
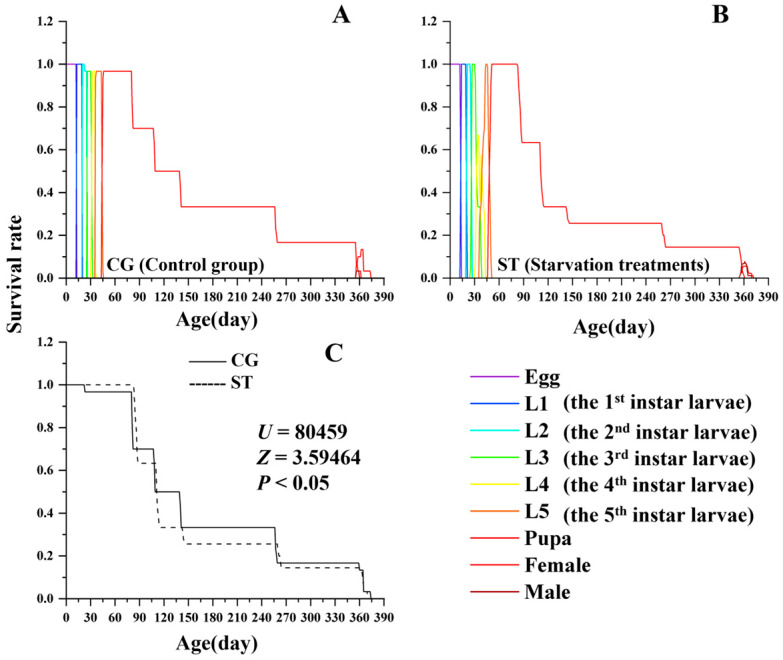
Comparison of age-specific survival rate (*l_x_*) and age-stage specific survival rate (*S_xj_*) between the control group (CG) and starvation treatments (ST) in *Luehdorfia chinensis*. (**A**) control group (CG); (**B**) starvation treatments (ST); (**C**) comparison between CG and ST.

Starvation also significantly altered the age-stage-specific survival curve (*l_x_*) of *L. chinensis*. As shown in Figure 1 (*U* = 80,459, *Z* = 3.59464, *p* < 0.05), both the starvation and control groups exhibited a Type III Deevey-type survival curve (*l_x_*), but the two curves differed significantly (*p* < 0.001). This discrepancy was mainly attributed to the pupal stage, during which the survival rate (*s_xj_*) in the starvation treatment was markedly lower than that of the control group.

### 3.2. Effects of Starvation on Adult Lifespan and Expectancy

**Figure 2:**
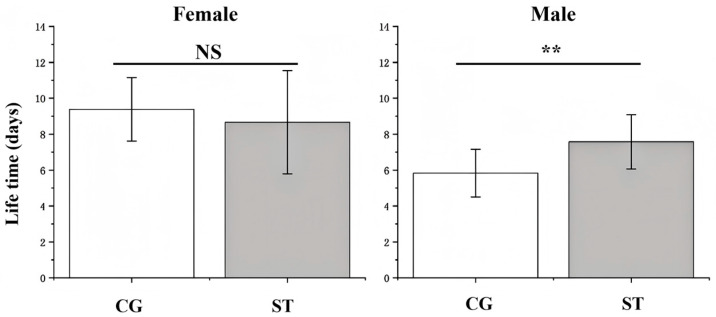
Effects of starvation on the fecundity and longevity of *Luehdorfia chinensis*. For adults: females—control group (CG), n = 8; starvation treatments (ST), n = 7; males—CG, n = 6; ST, n = 6. Data are presented as mean ± SE, NS, not significant (*p* > 0.05); **, significant difference at *p* < 0.01.

**Figure 3:**
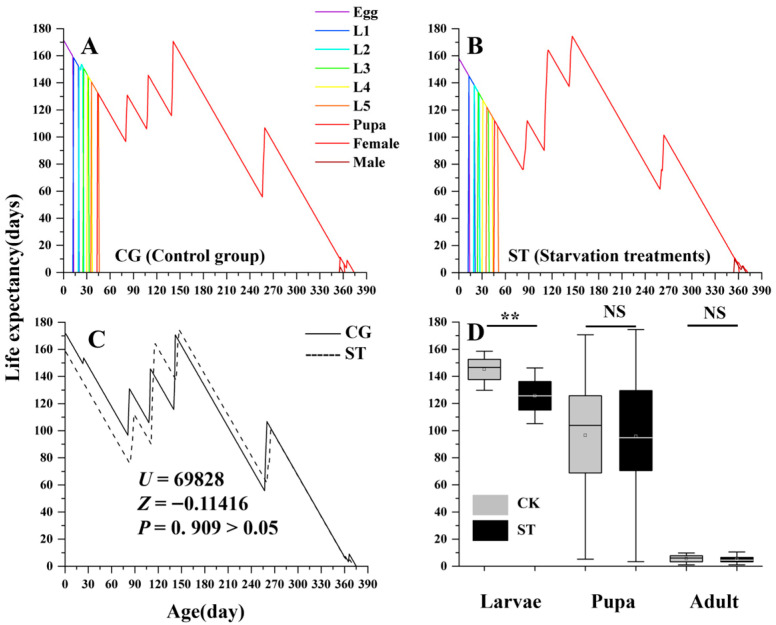
Comparison of age-stage specific life expectancy (*e_xj_*) between the control group (CG) and starvation treatments (ST) in *Luehdorfia chinensis*. (**A**) control group (CG); (**B**) starvation treatments (ST); (**C**) comparison between CG and ST; (**D**) stage-specific comparison (larval, pupa, adult) between CG and ST; data are presented as mean ± SE, NS, not significant (*p* > 0.05); **, significant difference at *p* < 0.01; The small square inside the box represents the mean value. Sample sizes for eggs, larval stages (L1–L5), and pupae are provided in Table 1. For adults: females—CG, n = 8; ST, n = 7; males—CG, n = 6; ST, n = 6.

The impact of starvation on adult lifespan varied between sexes. As shown in Figure 2, Female lifespan was not significantly affected by starvation, whereas male lifespan in the starvation treatment was significantly extended compared with the control. In contrast, the overall life expectancy (*e_xj_*) did not differ significantly between groups (Figure 3; *U* = 69,828, *Z* = −0.11416, *p* = 0 909 > 0.05). However, when assessed separately across developmental stages, the life expectancy of the larval stage exhibited a significant reduction in the starvation treatment, indicating negative effects on larval health and survival potential.

### 3.3. Effects of Starvation on Butterfly Fecundity

**Figure 4:**
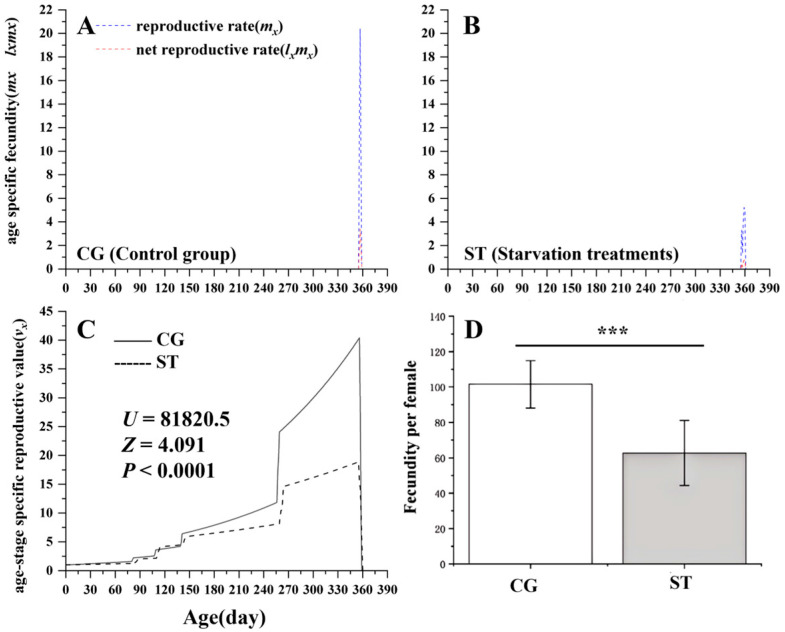
Comparison of age-specific fecundity (*m_x_*, *l_x_m_x_*) (**A**,**B**) and age-stage specific reproductive value (*v_x_*) (**C**) between the control group (CG) and starvation treatments (ST) in *Luehdorfia chinensis.* (**A**) control group (CG); (**B**) starvation treatments (ST); (**C**) comparison between CG and ST (*v_xj_*); (**D**) comparison of fecundity between CG females and ST females; data are presented as mean ± SE, ***, *p* < 0.001.

As illustrated in Figure 4 (*U* = 81,820.5, *Z* = 4.091, *p* < 0.0001), starvation significantly reduced the age-stage specific reproductive value (*v_x_*) of *L. chinensis* (*p* < 0.001). The age-specific fecundity (*m_x_*) of the starvation treatment was markedly lower (*m_x_* = 0.7) than that of the control group (*m_x_* = 21). Likewise, the net reproductive rate (*l_x_m_x_*) declined from 3.0 in the control group to 2.7 in the starvation treatment. Additionally, females that experienced larval starvation laid approximately 40% fewer eggs than control females, indicating a substantial decline in the reproductive output.

### 3.4. Effects of Starvation on Butterfly Population Dynamics

Starvation had a profound impact on the population dynamics of *L. chinensis*. As shown in Table 2, several key population parameters declined significantly in the starvation treatment. The intrinsic rate of increase (*r_m_*) decreased by 48.4%, the net reproductive rate (*R*_0_) declined by 59.5%, and total fecundity (*v_x_*) was reduced by 53.2%. In contrast, the finite rate of increase (*λ*) and mean generation time (*T*) remained relatively stable between groups.

**Figure 5:**
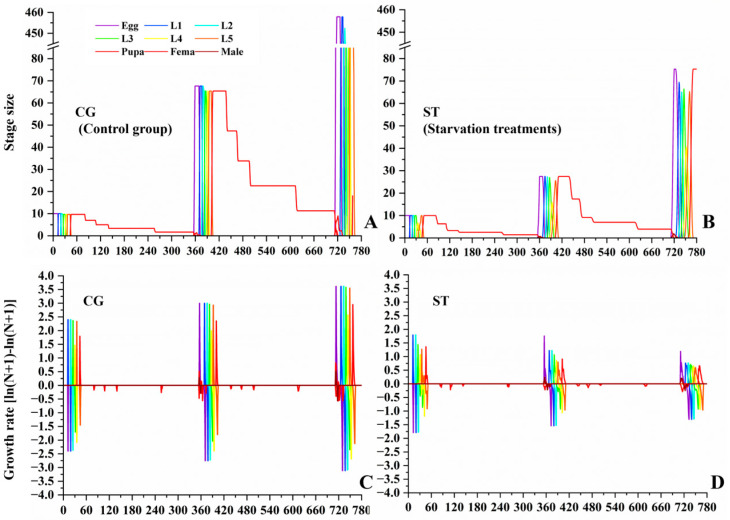
Comparison of stage size (**A**,**B**) and growth rate dynamics (**C**,**D**) between the control group (CG) and starvation treatments (ST) of *Luehdorfia chinensis*.

Based on the above population parameters, the butterfly population dynamic was projected for the next two years. As shown in Figure 5, the starvation treatment exhibited lower population growth, and the annual growth rate decreased from 1.7 in the first year to 1.3 in the second year. By the end of the second year, the predicted population size in the starvation treatment declined by more than 83% relative to the control group, suggesting a substantial risk of population decline under host-plant limitation.

## 4. Discussion

The larvae of the specialist butterfly *L. chinensis* exhibit a highly restricted diet, with different geographic populations feeding exclusively on either *A. sieboldii* or *A. forbesii* as their sole host plant [24,25]. Consequently, the occurrence and distribution patterns of this species in the wild are strongly dependent on the abundance and spatial distribution of host plants [25,35,55]. In areas where host resources are limited, adults may face oviposition constraints, while larvae are prone to starvation stress, potentially leading to greater population fluctuations and an elevated risk of local extinction [34,56,57]. In this study, we conducted a controlled starvation experiment to simulate the population dynamics of *L. chinensis* under host plant limitation, as observed in wild populations such as those in Taohongling of Jiangxi Province. Our results showed that third- to fifth-instar larvae exposed to three days of starvation exhibited significantly prolonged developmental periods, reduced fecundity, and marked declines in multiple fitness-related parameters, including the intrinsic rate of increase (*r_m_*). Model projections further suggest that starvation stress could lead to a population decline exceeding 83% within the following two years. These findings provide direct empirical evidence supporting the hypothesis that host plant limitation drives butterfly population decline and underscore the critical importance of maintaining and restoring key habitat resources in the conservation of endangered butterflies [18,58].

The persistence of butterfly populations in the wild depends on two main categories of habitat resources: (1) consuming resources, such as host plants and nectar sources; and (2) utilities, including functional sites for courtship, mating, and pupation [18,58]. For the monophagous *L. chinensis*, the host plants *A. sieboldii* or *A. forbesii* are indispensable for population maintenance [25,35,55]. However, over the past decades, species of *Asarum* have suffered extensive harvesting and habitat destruction due to their medicinal value [35,38,39]. These perennial plants grow slowly, have strict habitat requirements (e.g., shaded, moist, fertile soils), and exhibit poor natural recovery, especially under adverse conditions such as climate warming and frequent extreme weather events [59]. As a result, remnant host plant resources remain scarce, placing many *L. chinensis* populations under chronic oviposition and foraging constraints. Unlike naturally occurring resource fluctuations, starvation stress driven by anthropogenic disturbance or long-term ecological change tends to be frequent and persistent, with potentially severe consequences for both individuals and populations [60].

During starvation, the balance between mass and energy flux through an animal becomes disrupted [60]. For insects such as butterflies, the imbalance of endogenous substances and energy caused by larval starvation may eventually lead to a reduction in individual fitness [61]. Generally, the response of insects such as butterflies to starvation stress is more complex. On one hand, larvae may prolong development and feeding periods to compensate for nutritional deficits and accumulate energy for metamorphosis. For example, larvae of *Bicyclus anynana* (Butler, 1879) (Lepidoptera: Nymphalidae) significantly extend their developmental duration under dietary restriction to increase feeding time [62]. In the present study, *L. chinensis* larvae subjected to starvation similarly prolonged each instar, likely as an energy compensation strategy, a pattern consistent with previous observations in other caterpillars. On the other hand, the pupal period was shortened, leading to earlier adult emergence. This may reflect an energy reallocation mechanism, in which extending feeding stages enhances energy accumulation, while compressing non-feeding stages reduces mortality risk [63,64]. Similar patterns have been reported in other Lepidoptera, where larval starvation generally prolongs development and reduces pupal mass or fecundity [65,66,67]. Notably, a shortened pupal duration has also been observed in starved males of *Lycaena tityrus* (Poda, 1761) (Lepidoptera: Lycaenidae), indicating that such a response could occur in other species of Lepidoptera [24]. In addition, starvation markedly suppressed the adult reproductive capacity. According to a common survival trade-off mechanism, insects tend to reduce reproductive investment in response to starvation in order to maintain somatic functions [68,69,70]. In *L. chinensis*, larval starvation significantly reduced female egg production, a pattern also reported in *Pieris brassicae* (Linnaeus, 1758) (Lepidoptera: Pieridae) and *Hyphantria cunea* (Drury, 1773) (Lepidoptera: Arctiidae) [65,71]. Given that *L. chinensis* adults have limited feeding capacity, reproductive system development relies heavily on energy reserves acquired during the larval stage [72], suggesting that starvation may force individuals to prioritize basic survival over reproduction [70,73,74].

Host plant limitation–induced larval starvation not only reduces individual fitness but also constrains population growth potential [20,65]. In this study, the *r_m_*, net reproductive rate (*R*_0_), and total fecundity (*v_x_*) of the starvation group were all significantly lower than those of the control group. Population modeling predicted that continued starvation could lead to a population decline exceeding 83% within two years. For endangered taxa such as *L. chinensis*, persistent population decline can exacerbate the effects of genetic drift and inbreeding depression, resulting in the progressive erosion of allelic diversity and a concomitant reduction in adaptive potential to environmental variability. According to the extinction vortex framework [75,76,77], these genetic processes interact synergistically with ecological stochasticity, demographic stochasticity, and maladaptive behavioral responses, creating self-reinforcing feedback loops that accelerate population decline toward extinction. For instance, *Euphydryas aurinia* (Rottemburg, 1775) (Lepidoptera: Nymphalidae) has experienced increased subpopulation instability and elevated extinction risk due to the loss of its larval host plant (*Succisa pratensis* Moench) and habitat fragmentation [78], while western North American monarch butterflies (*Danaus plexippus* (Linnaeus, 1758) (Lepidoptera: Nymphalidae)) have declined from millions in the 1980s to fewer than 2000 individuals in 2020 under the combined pressures of pesticide use, habitat loss, and climate change [79]. These cases highlight the universality and severity of multiple interacting factors accelerating butterfly population decline and further underscore the central importance of maintaining and restoring critical habitat resources in the conservation of endangered butterflies [80,81].

## 5. Conclusions

The survival of the endangered butterfly *L. chinensis* is tightly linked to the availability of its host plants (*A. sieboldii* or *A. forbesii*), making local populations highly vulnerable to host plant limitation. In the present study, we simulated such resource constraints in the wild by subjecting larvae to short-term starvation stress. Our results demonstrated that starvation not only impaired larval growth and development but also exerted delayed effects on subsequent life stages, including the pupal and adult phases. In particular, female fecundity was markedly reduced, leading to lower individual fitness and, consequently, a decline in population size. These findings provide novel evidence for the hypothesis that host plant limitation is a key driver of population decline in *L. chinensis*, contributing to the broader understanding of global butterfly declines [8,9,10]. Furthermore, they emphasize the necessity of conserving and restoring critical habitat resources as a core component of butterfly conservation programs, and highlight the applicability of the “resource-based habitat” concept to the protection of specialist species such as *L. chinensis* [11,60].

## Figures and Tables

**Table 1:** Effects of starvation on the developmental duration (Mean ± SD) of immature stages in *Luehdorfia chinensis*.

Stages	CG (n)	Starvation Treatments (ST)			Kruskal–Wallis Test
		3rd (n)	4th (n)	5th (n)	
egg	13.00 ± 0.00 a (30)	13.97 ± 0.18 a (30)	13.33 ± 0.48 a (30)	13.20 ± 0.41 a (30)	*χ*^2^ = 6.86538, *df* = 3, *p* = 0.07631
1st instar larvae	7.00 ± 0.00 a (30)	7.00 ± 0.00 a (30)	7.00 ± 0.00 a (30)	7.00 ± 0.00 a (30)	*χ*^2^ = 0, *df* = 3, *p* = 1
2nd instar larvae	6.00 ± 0.00 a (30)	6.00 ± 0.00 a (30)	5.83 ± 0.38 a (30)	6.00 ± 0.00 a (30)	*χ*^2^ = 6.40, *df* = 3, *p* = 0.0938
3rd instar larvae	5.69 ± 0.10 b (30)	11.60 ± 0.40 a (30)	5.47 ± 0.51 b (30)	5.73 ± 0.57 b (30)	*χ*^2^ = 86.39, *df* = 3, *p* < 0.0001
4th instar larvae	4.34 ± 0.09 b (30)	4.77 ± 0.43 b (30)	7.03 ± 0.18 a (30)	4.33 ± 0.48 b (30)	*χ*^2^ = 83.23, *df* = 3, *p* < 0.0001
5th instar larvae	8.48 ± 0.09 b (30)	7.20 ± 0.41 c (30)	8.37 ± 0.49 b (30)	11.23 ± 0.41 a (30)	*χ*^2^ = 100.83, *df* = 3, *p* < 0.0001
Larval duration	31.37 ± 0.76 c (30)	35.93 ± 0.64 a (30)	33.77 ± 0.90 b (30)	33.97 ± 0.72 b (30)	*χ*^2^ = 99.97, *df* = 3, *p* < 0.0001
Pupa	313.2 ± 0.97 a (14)	308.14 ± 0.71 b (6)	309.00 ± 0.00 b (2)	308.75 ± 0.85 b (5)	*χ*^2^ = 9.83, *df* = 3, *p* < 0.05
Pre-adult emergence (Female)	365.20 + 3.56 a (8)	359.5 ± 0.5 b (2)	358 (1)	356.33 + 2.33 b (3)	*χ*^2^ = 8.79, *df* = 2, *p* < 0.05
Pre-adult emergence (Male)	365.75 + 3.41 a (6)	357.4 + 4.8 b (4)	358 (1)	356.50 + 1.36 b (2)	*χ*^2^ = 7.02, *df* = 2, *p* < 0.05

Note: Different letters within a row indicate significant differences (Kruskal–Wallis test, Dunn’s post hoc, *p* < 0.05); values with n = 1 are shown without SD; pre-adult emergence (female and male) was compared only among CG, 3rd, and 5th instar treatments.

**Table 2:** Comparison of population parameters (Mean ± SD) between the control group (CG) and starvation treatments (ST) in *Luehdorfia chinensis*.

Population Parameters	CG (n = 30)	ST (n = 90)
Intrinsic rate of increase (*r*)	0.0064 ± 0.0026 a	0.0033 ± 0.0016 b
Net reproductive rate (*R*_0_)	6.77 ± 4.72 a	2.74 ± 1.4 b
Finite rate of increase (*λ*)	1.0064 ± 0.0027 a	1.0033 ± 0.0017 a
Total fecundity (*F*)	40.6 ± 27.53 a	19 ± 8.92 b
Mean generation time (*T*)	357.87 ± 0.69 a	358.66 ± 0.84 a

Note: Data in the table are compared horizontally, with different letters representing differences between groups at a significance level of *p* < 0.05.

## Data Availability

The original contributions presented in this study are included in the article. Further inquiries can be directed to the corresponding author.

## Supplementary Materials

The following supporting information can be downloaded at: https://www.mdpi.com/article/10.3390/insects16100995/s1. Figure S1: Laboratory setup; Figure S2: Rearing container.
