# Supplementary material for: Starvation During the Larval Stage Driving Population Decline in the Butterfly Specialist Luehdorfia chinensis Leech, 1893 (Lepidoptera: Papilionidae)
Source: Insects. 2025 Sep 24;16(10):995. doi: 10.3390/insects16100995 (PMC12564415; doi:10.3390/insects16100995)
Supplement: Supplementary file 1 [file insects-16-00995-s001.zip › insects-3850273-supplementary.pdf]

insects

MDPI

Article

Starvation During the Larval Stage Driving Population Decline in the Butterfly Specialist *Luehdorfia chinensis* Leech, 1893 (Lepidoptera: Papilionidae)

Wenjing Yang [1,2], Qi Zhu [1,2], Yunhao Zou [1,2], Chao Yang [3], Wenguo Wu [4], Qin Zou [2] and Juping Zeng [1,2,*]

1 Jiangxi Provincial Key Laboratory of Conservation Biology, Key Laboratory of National Forestry and Grass and Administration on Forest Ecosystem Protection and Restoration of Poyang Lake Watershed, College of Forestry, Jiangxi Agricultural University, Nanchang 330045, China; yangwenjing9351@163.com (W.Y.); 18870789330@163.com (Q.Z.); zouyunhao@stu.jxau.edu.cn (Y.Z.)
2 Lushan Forest Ecosystem Observation Station, Lushan National Nature Reserve of Jiangxi, Jiujiang 332900, China; zouqin496141285@163.com
3 Lushan Forestry Bureau of Jiangxi, Lushan 332800, China; 15305193314@163.com
4 Taohongling Sika Deer National Nature Reserve of Jiangxi, Pengze 332724, China; wuwenguo123@126.com
* Correspondence: zengjupingjxau@163.com. Tel.: +86-15180428842; Fax.: +86-791-83828039

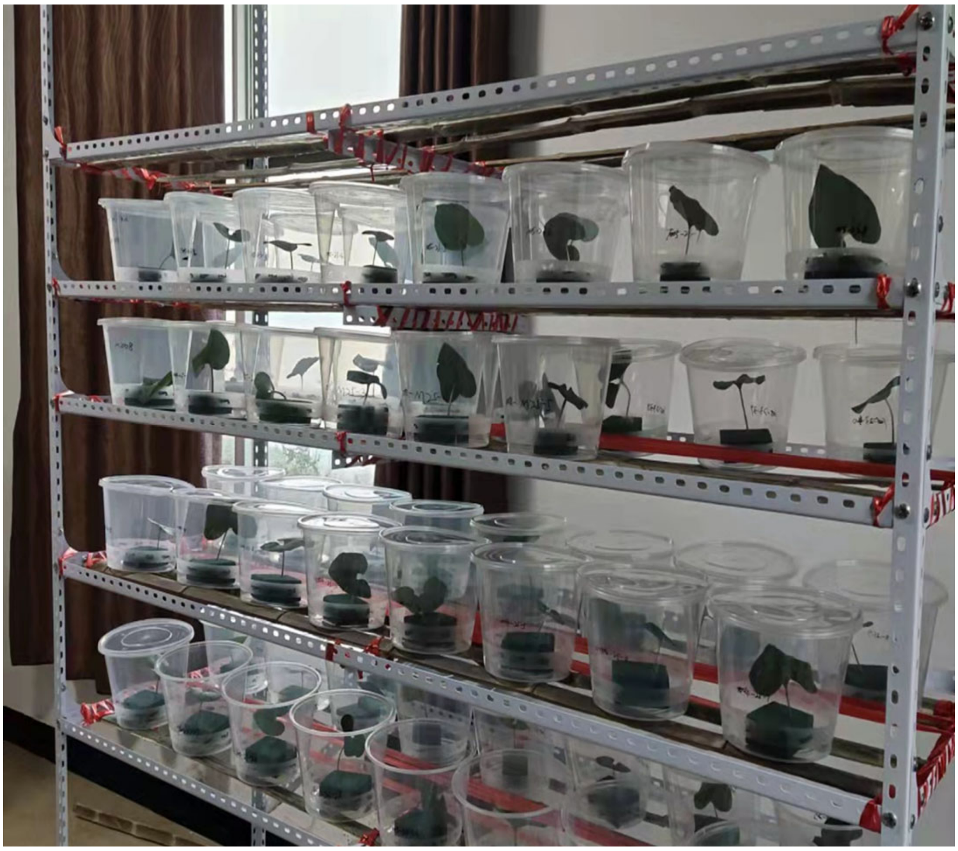

Figure S1. Laboratory setup.

 https://doi.org/10.3390/insects16100995

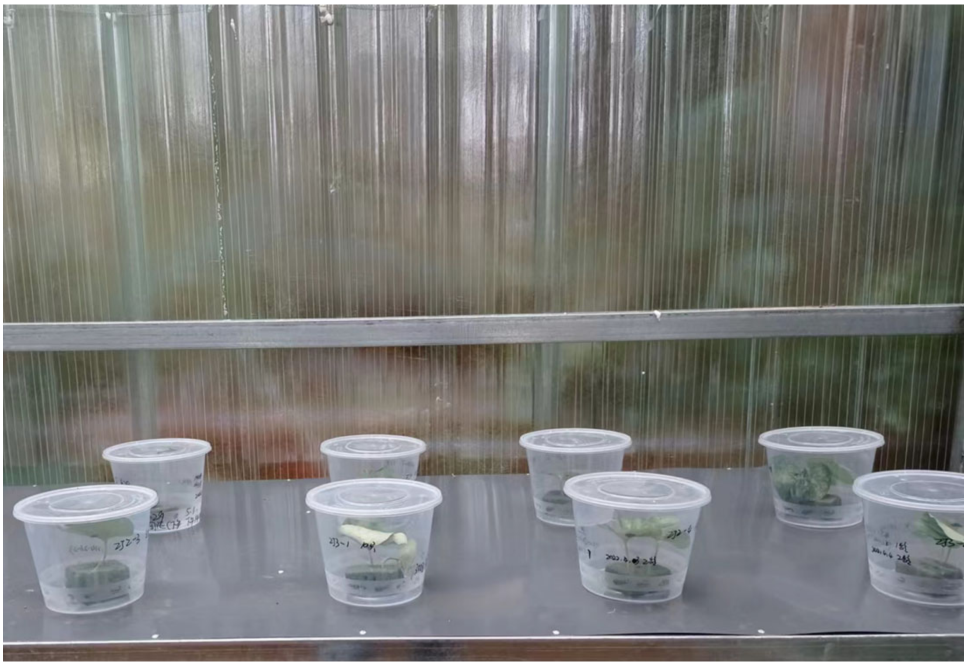

Figure S2. Rearing_container.
